# The Prognostic Significance of Functional Mitral Regurgitation in Patients with Cardiovascular–Kidney–Metabolic Syndrome

**DOI:** 10.3390/jcm15072679

**Published:** 2026-04-01

**Authors:** Haodong Du, Yangyang Chen, Chunlu Huang, Botao Hu, Zhe Wang, Shuai Shao, Qiankun Bao, Ya Suo

**Affiliations:** 1 Tianjin Key Laboratory of Ionic-Molecular Function of Cardiovascular Disease, Department of Cardiology, Tianjin Institute of Cardiology, The Second Hospital of Tianjin Medical University, Tianjin 300211, China; duhaodongdoc@tmu.edu.cn (H.D.); chenyangyang@tmu.edu.cn (Y.C.); suaner5664@tmu.edu.cn (C.H.); hubotao@tmu.edu.cn (B.H.); shaoshuai0712@tmu.edu.cn (S.S.); 2 Department of Kidney Disease and Blood Purification Centre, The Second Hospital of Tianjin Medical University, Tianjin 300211, China; cmu_wz@hotmail.com

**Keywords:** cardiovascular–kidney–metabolic syndrome, functional mitral regurgitation, atrial functional mitral regurgitation, heart failure with preserved ejection fraction, end-stage renal disease

## Abstract

**Background:** Cardiovascular–kidney–metabolic (CKM) syndrome delineates the occurrence of clinical cardiovascular diseases alongside renal or metabolic complications. The prognostic implications of functional mitral regurgitation (FMR) in patients with CKM syndrome remain unclear. This study aimed to elucidate the association and independent impact of FMR regarding clinical outcomes in this population. **Methods:** In this retrospective cohort study, 1201 patients with CKM syndrome undergoing maintenance hemodialysis between January 2020 and November 2024 were analyzed. Participants were stratified according to the presence and severity of FMR/atrial functional mitral regurgitation (AFMR) into the non-FMR/AFMR, mild FMR/AFMR, and moderate-to-severe FMR/AFMR groups. The primary outcome measure was all-cause mortality. Survival analyses were performed using Kaplan–Meier estimates and multivariate Cox proportional hazard models. **Results:** FMR prevalence was higher (79.68%) in the CKM cohort. FMR was independently associated with increased all-cause mortality (hazard ratio [HR], 1.831; 95% confidence interval [CI], 1.320–2.541; *p* = 0.0019). Even mild FMR conferred a significantly elevated risk compared to patients without FMR (HR, 1.699; 95% CI, 1.200–2.405; *p* = 0.0078). Similarly, AFMR was an independent predictor of mortality (HR, 2.106; 95% CI, 1.349–3.289; *p* = 0.0046), with moderate-to-severe AFMR associated with a three-fold higher risk (HR, 3.294; 95% CI, 1.007–10.78; *p* = 0.0027) than non-AFMR. **Conclusions:** Both FMR and AFMR were found to be independently associated with elevated all-cause mortality in patients with CKM syndrome undergoing hemodialysis. These findings underscore the critical need for early detection and tailored management of mitral regurgitation in this high-risk population to potentially improve outcomes.

## 1. Introduction

Cardiovascular–kidney–metabolic (CKM) syndrome is a systemic disorder characterized by pathophysiological interactions among metabolic risk factors, chronic kidney disease (CKD), and cardiovascular disease; the syndrome often culminates in multi-organ dysfunction and an elevated risk of adverse cardiovascular outcomes [1]. The underlying pathophysiology involves a complex interplay of hemodynamic and neurohormonal mechanisms, including sympathetic overactivation, renin–angiotensin–aldosterone system dysregulation, imbalances in vasoactive mediators (e.g., nitric oxide and endothelin), and oxidative stress [2,3]. The clinical staging of CKM syndrome enables effective stratification of its cardiovascular mortality risk. For instance, data from a nationally representative cohort of U.S. adults demonstrated a graded increase in cardiovascular mortality across advanced CKM stages (1–4), with stage 4 associated with a 3.88-fold higher risk than stage 0 [4,5]. The risk is particularly pronounced in patients with end-stage renal disease (ESRD) on maintenance hemodialysis who, according to the American Heart Association (AHA) criteria, are classified as having CKM stage 4b and experience significantly high cardiovascular mortality rates [6].

Mitral regurgitation (MR) is a common valvular heart disease, affecting approximately 2.5% of the population, and is independently associated with increased hospitalization for heart failure (HF) and mortality [7]. Functional mitral regurgitation (FMR) is distinguished from primary (organic) mitral valve disease based on the absence of an intrinsic leaflet pathology. This arises from an imbalance between the tethering forces on the mitral apparatus and the left ventricular closure forces, leading to inadequate leaflet coaptation. Based on the predominant underlying cardiac remodeling, FMR is further subdivided into atrial functional mitral regurgitation (AFMR) and ventricular functional mitral regurgitation (VFMR) [8]. AFMR primarily results from left atrial dilatation, often in the context of atrial fibrillation (AF) and/or HF with preserved ejection fraction (HFpEF), and is frequently observed in elderly patients with HF [9]. In contrast, VFMR is typically driven by left ventricular dilation and systolic dysfunction, which are commonly associated with ischemic or dilated cardiomyopathy [10].

A substantial body of evidence links FMR to worse outcomes in patients with cardiovascular, renal, and metabolic diseases. Registry data indicate that even mild FMR at discharge is associated with an adverse prognosis in patients hospitalized for acute decompensated HF [11]. The burden of FMR is significantly higher in patients with CKD (42.9%) than in the general population (23.8%), and its presence confers a >50% increase in 5-year mortality among patients with valvular heart disease [12]. Similarly, in type 2 diabetes mellitus (T2DM), concomitant FMR is linked to a markedly elevated risk of all-cause mortality, ranging from 3.3-fold for mild FMR to 5.1-fold for moderate-to-severe FMR [13].

The integrative concept of CKM syndrome provides a coherent framework for studying the confluence of metabolic, renal, and cardiovascular pathologies [1]. However, the specific prevalence of FMR, particularly its atrial subtype (AFMR), within the population with CKM and its impact on clinical outcomes, such as all-cause mortality, remain poorly characterized. Therefore, this study aimed to investigate the prevalence of FMR and AFMR in patients with CKM syndrome and evaluate their association with all-cause mortality.

## 2. Methods

### 2.1. Study Design and Participants

This study was conducted at the Hemodialysis Center of the Second Hospital of Tianjin Medical University. The study protocol was approved by the hospital Medical Ethics Committee and written informed consent was obtained from all participants.

We initially identified 1627 patients with ESRD and HFpEF who were diagnosed with CKM syndrome and received maintenance hemodialysis between 3 January 2020 and 22 November 2024. HFpEF was defined according to the 2021 ESC Guidelines as the presence of symptoms or signs of HF, evidence of structural/functional cardiac abnormalities and/or elevated natriuretic peptides, and left ventricular ejection fraction (LVEF) ≥ 50% [14]. CKM syndrome is staged according to the AHA classification system, which incorporates cardiovascular, renal, and metabolic risk components [1]. Demographic, clinical, echocardiographic, laboratory, and outcome data were collected for 1627 patients, all of whom met the criteria for CKM stage 4b. Of these, 202 participants were excluded because of dialysis duration < 3 months, survival time < 1 month, or incomplete clinical and echocardiographic data. Among the remaining 1425 individuals, an additional 224 were excluded for the following reasons: (1) structural mitral valve pathology or moderate-to-severe aortic valve disease; (2) left ventricular outflow tract obstruction; (3) history of kidney transplantation or cardiac surgery; (4) constrictive pericarditis or congenital heart disease, resulting in a final cohort of 1201 patients.

**Figure 1:**
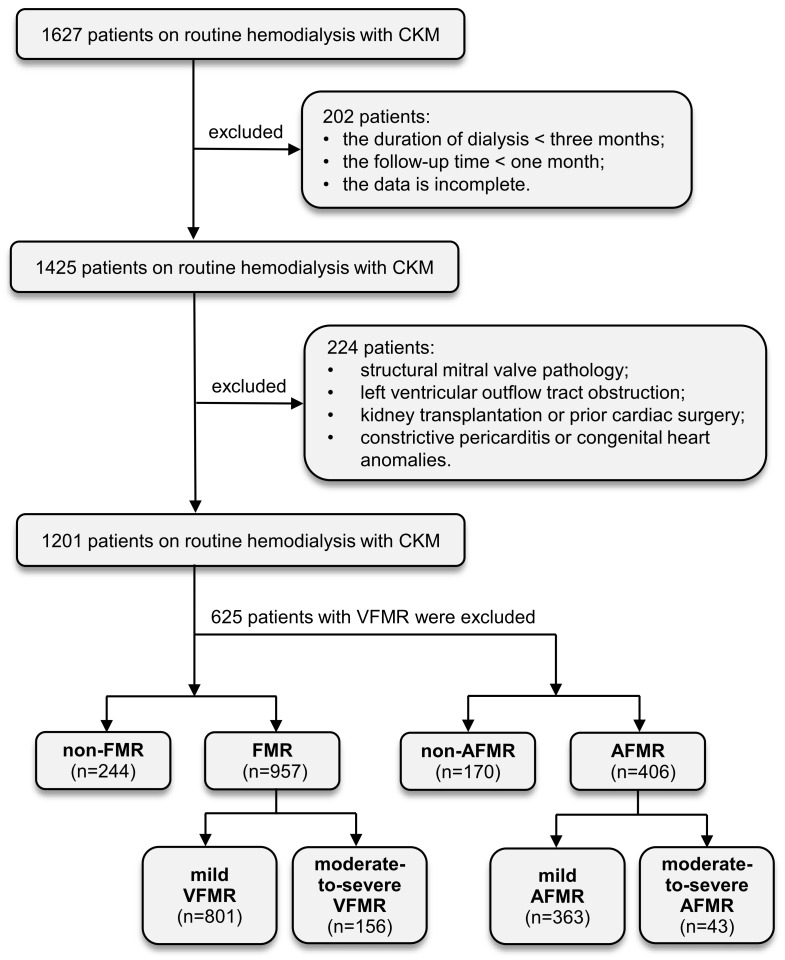
Study design flowchart; 1201 patients enrolled in this study underwent data analysis. Abbreviations: CKM, Cardiovascular–Kidney–Metabolic syndrome; FMR, functional mitral regurgitation; VFMR, ventricular functional mitral regurgitation; AFMR, Atrial Functional Mitral Regurgitation.

Finally, 1201 patients were included in the final analysis. The median follow-up period was 30 months (interquartile range [IQR], 13–43 months; range, 1–61 months). The patients underwent conventional hemodialysis (three sessions weekly, 4 h per session). Transthoracic echocardiography was generally performed on a mid-week interdialytic day. Based on regurgitant volume, patients were classified into a non-FMR group (*n* = 244) and an FMR group (*n* = 957). The FMR group was further stratified into mild (*n* = 801) and moderate-to-severe (*n* = 156) FMR subgroups. Subsequently, 625 patients with VFMR, defined as left ventricular dilation and/or segmental wall motion abnormalities, were excluded. The remaining patients with AFMR were divided into non-AFMR (*n* = 170) and AFMR (*n* = 406) cohorts. The median follow-up period was 36 months (IQR, 18–49 months; range, 1–61 months). The AFMR cohort was further subdivided into mild (*n* = 363) and moderate-to-severe (*n* = 43) subgroups. The date of the first echocardiographic assessment defining the AFMR severity of AFMR was designated as the start of the observation period. A flowchart of the patient selection process is shown in Figure 1.

### 2.2. Data Collection

Epidemiological, demographic, survival, clinical, and laboratory data were extracted from electronic medical records. Standard laboratory assessments, including routine blood tests and renal function evaluations, were performed in all patients before dialysis initiation.

### 2.3. Transthoracic Echocardiography (TTE)

All transthoracic echocardiographic examinations during the study period were performed in the echocardiography laboratory of Tianjin Medical University Second Hospital. This setting enabled the comprehensive identification of patients diagnosed with FMR and AFMR using Doppler echocardiography by reviewing archived digital images and electronically stored data. All images were independently reviewed and interpreted by senior cardiologists with substantial expertise in echocardiography, who were blinded to the results of other clinical investigations.

The diagnostic assessment of FMR, VFMR, and AFMR followed an integrated standardized protocol incorporating qualitative, semi-quantitative (e.g., vena contracta width, jet/LA ratio), and quantitative (e.g., regurgitant volume, regurgitant fraction, effective regurgitant orifice area, as available) measures to grade regurgitation severity, as referenced in established guidelines [15]. The severity of FMR was evaluated primarily using regurgitant volume calculated via the continuity equation (none: 0 mL, mild: <30 mL, moderate: 30–59 mL, severe: ≥60 mL) in a consistent manner across the study [16].

Standard echocardiographic views and techniques were used to obtain the following parameters: left atrial diameter (LAD), left ventricular ejection fraction (LVEF), interventricular septal thickness (IVST), left ventricular internal diameter at end-diastole (LVIDd), left ventricular posterior wall thickness (LVPW), ratio of early diastolic transmitral velocity to early diastolic mitral annular tissue velocity (E/e’), mitral annular calcification (MAC) and pulmonary artery systolic pressure. Pulmonary hypertension (PH) was defined as an estimated pulmonary artery systolic pressure exceeding 35 mmHg at rest.

### 2.4. Statistical Analysis

Demographic and clinical characteristics, along with laboratory indices, were reported as means (standard deviation) or number (percentage). Categorical variables were analyzed between groups using the chi-square test, and continuous variables were assessed using one-way ANOVA or a two-tailed *t*-test. A *p* value < 0.05 was deemed statistically significant. The log-rank test and Kaplan–Meier survival curves were used to assess the clinical all-cause mortality with or without FMR. Cox regression models were used to conduct univariate and multivariate analyses of mortality, considering deaths with or without FMR. Cox regression models were used to examine the impact of the FMR on primary outcome. Predefined potential confounding variables were included in the univariate analyses along with FMR. Variables with a *p* value < 0.05 in univariable analysis were incorporated into the multivariable analysis using a forced entry method. To assess the robustness of the Cox proportional hazards model, the proportional hazards assumption was tested for all covariates using Schoenfeld residuals. The analysis was performed using the survival package in the R software. The C-index was calculated using the survConcordance function in R and its 95% confidence interval (CI) was computed based on standard errors.

All statistical analyses were conducted using SPSS software (version 26.0) and R software (version 4.4.3). Graphs were generated using GraphPad Prism (version 10.2).

## 3. Results

### 3.1. Clinical, Echocardiographic, and Laboratory Profiles of the Study Cohort

The baseline clinical characteristics of the 1201 participants are summarized in Table 1. The mean age was 56.58 ± 13.94 years, and 695 (57.87%) were male. Coronary heart disease (CHD), hypertension, and diabetes mellitus were the most common comorbidities. Specifically, 569 patients (47.50%) had CHD, 1123 (93.51%) had hypertension, 479 (39.92%) had diabetes mellitus, 39 (3.25%) had a history of myocardial infarction (MI), 218 (18.15%) had a history of stroke, 40 (3.33%) had AF, and 184 (15.32%) had PH. FMR was present in 957 patients (79.7%). Compared to the non-FMR group (*n* = 244), patients with FMR had significantly higher proportions of CHD, diabetes, and PH, suggesting potential pathophysiological links between these conditions and FMR. Echocardiographic assessment revealed that the FMR group had a significantly larger LAD, LVIDd, IVST, and LVPW, as well as a higher E/e’ ratio, MAC prevalence, and tricuspid regurgitation severity, indicative of left heart enlargement and diastolic dysfunction. Although all patients had HFpEF, LVEF was significantly lower in the FMR group, suggesting a mild impairment in systolic function. Laboratory analysis demonstrated that N-terminal pro-B-type natriuretic peptide (NT-proBNP) levels were markedly higher in patients with FMR, whereas no significant intergroup differences were observed in serum creatinine, blood urea nitrogen, or calcium-phosphorus levels. Furthermore, patients with FMR exhibited significantly lower levels of circulating triglycerides and very-low-density lipoprotein (VLDL) (Table 1 and Table A1).

### 3.2. FMR Independently Predicts All-Cause Mortality in CKM

**Figure 2:**
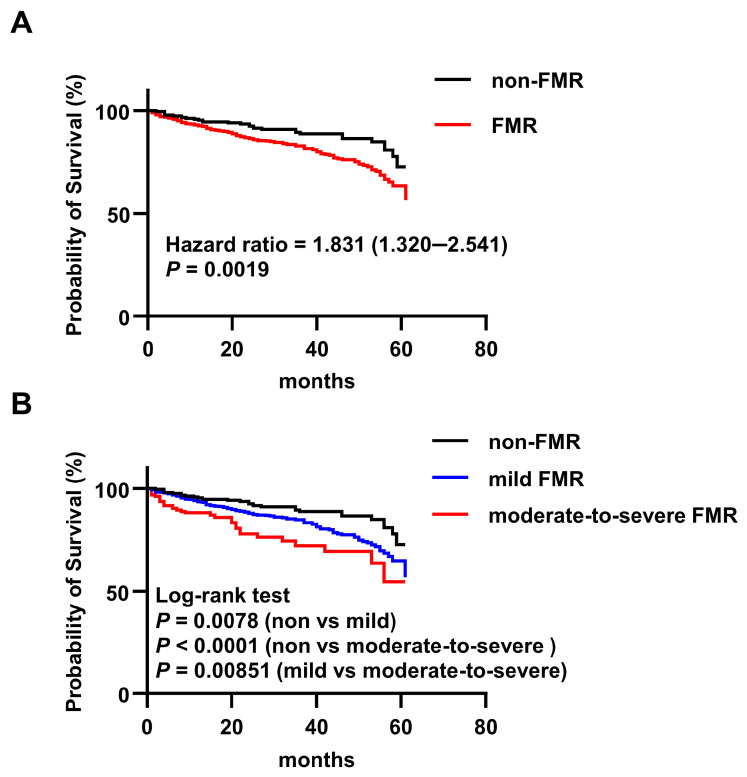
Kaplan–Meier analysis of all-cause mortality in patients with FMR and CKM. (**A**) Kaplan–Meier survival curves were generated for patients with CKM syndrome with (*n* = 957) or without (*n* = 244) functional mitral regurgitation (FMR). (**B**) Kaplan–Meier survival curves were generated for patients with CKM syndrome among non-FMR (*n* = 244), mild FMR (*n* = 801) and moderate–severe FMR (*n* = 156).

Patients were stratified according to the presence or absence of FMR (non-FMR, *n* = 244; FMR, *n* = 957). Over a median follow-up period of 30 months, 185 all-cause deaths occurred, including 30 (16.2%) in the non-FMR group and 155 (83.8%) in the FMR group. To further characterize the causes of death, we examined cardiovascular mortality in patients with known causes of death. Of the 185 deceased patients, 58 were excluded due to unclear causes of death based on electronic medical records. Among the remaining 127 patients, cardiovascular deaths accounted for 65.2% (15/23) in the non-FMR group, 87.7% (71/81) in the mild FMR group, and 91.3% (21/23) in the moderate-to-severe FMR group, indicating a progressively higher proportion of cardiovascular deaths with increasing FMR severity. Kaplan–Meier analysis with the log-rank test demonstrated a significant association between FMR and all-cause mortality. The estimated 5.95-year survival rates were 87.7% for non-FMR patients and 83.8% for FMR patients (HR, 1.831; 95% CI, 1.320–2.541; *p* = 0.0019) (Figure 2A). Univariate Cox regression analysis identified age, CHD, previous myocardial infarction, diabetes, AF, stroke, creatinine, red blood cell count, platelet count, calcium, glucose, and FMR as factors associated with mortality. In a multivariable Cox model adjusted for these covariates, FMR remained independently associated with an increased risk of all-cause mortality (HR, 1.64; 95% CI, 1.09–2.48; *p* = 0.019) (Table 2). The proportional hazards assumption was tested using Schoenfeld residuals; it was satisfied for FMR (*p* = 0.721). The multivariable model demonstrated good discrimination for all-cause mortality, with a Harrell’s C-index of 0.720 (95% CI, 0.676–0.763).

### 3.3. Association Between FMR Severity and Mortality Risk

Patients were further classified into mild FMR and moderate-to-severe FMR subgroups based on regurgitant volume. The clinical characteristics of the patients are shown in Table 3 and Table A2. Compared with the non-FMR and mild FMR groups, patients with moderate-to-severe FMR had significantly greater LAD, LVIDd, E/e’ ratio, and NT-proBNP levels, suggesting progressive left heart remodeling and diastolic dysfunction with increasing regurgitation severity. The prevalence of MAC was highest in the moderate-to-severe FMR group. Survival analysis revealed that patients with mild FMR had a higher mortality risk than non-FMR patients (5.95-year survival, 84.5% vs. 87.7%; HR, 1.699; 95% CI, 1.200–2.405; *p* = 0.0078). The risk was further elevated in patients with moderate-to-severe FMR compared to those with mild FMR (5.95-year survival, 80% vs. 84.5%; HR, 1.672; 95% CI, 1.054–2.651; *p* = 0.0085) (Figure 2B).

### 3.4. Echocardiographic and Laboratory Correlates of FMR Severity

**Figure 3:**
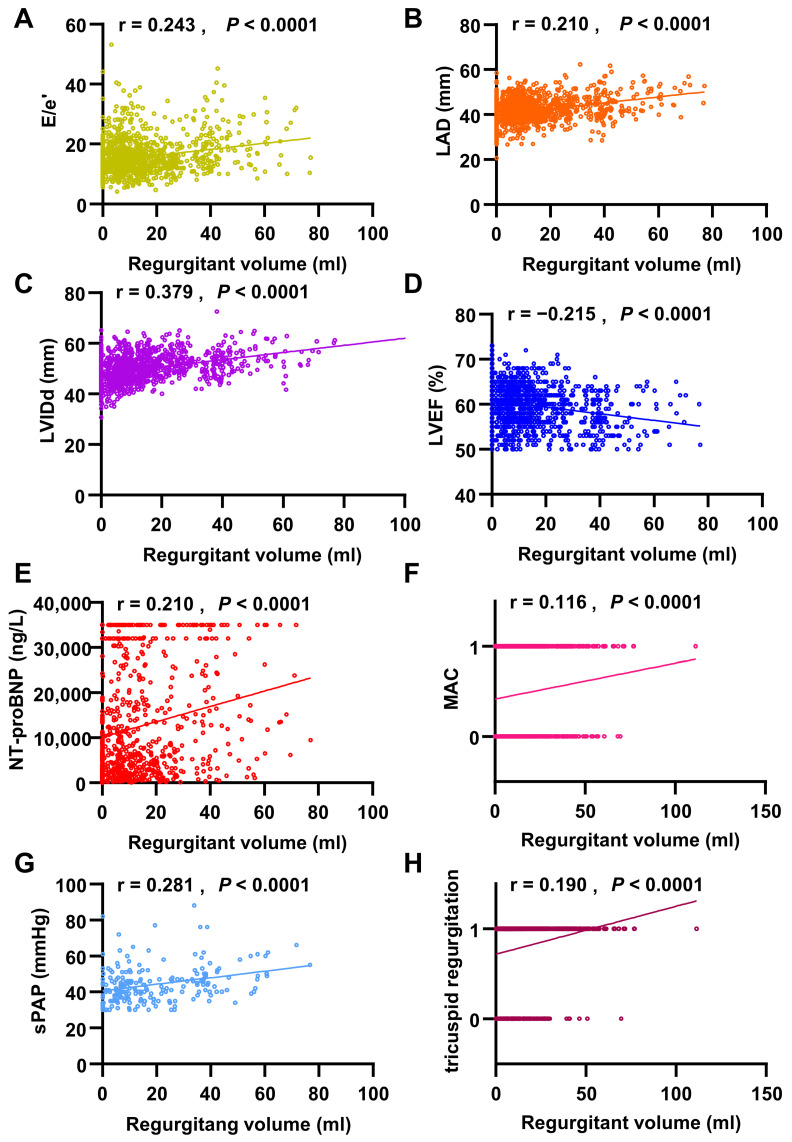
The relationships among echocardiographic parameters, NT-proBNP levels, and FMR in patients with CKM syndrome. (**A**) The E/e’ ratio (r = 0.243) is positively correlated with regurgitating volume. (**B**) The LAD (r = 0.210) is positively correlated with regurgitating volume. (**C**) The LVIDd (r = 0.379) is positively correlated with regurgitating volume. (**D**) The LVEF (r = −0.215) is negatively correlated with regurgitating volume. (**E**) The NT-proBNP level (r = 0.210) is positively correlated with regurgitating volume. (**F**) The MAC (r = 0.116) is positively correlated with regurgitating volume. (**G**) The systolic pulmonary artery pressure (sPAP) (r = 0.281) is positively correlated with regurgitating volume. (**H**) The tricuspid regurgitation (r = 0.190) is positively correlated with regurgitating volume.

Our retrospective investigation verified that the FMR is independently associated with an increased risk of all-cause mortality. FMR was primarily diagnosed using transthoracic echocardiography. Linear regression analysis revealed that regurgitant volume was positively correlated with LAD (r = 0.210, *p* < 0.0001), LVIDd (r = 0.379, *p* < 0.0001), E/e’ ratio (r = 0.243, *p* < 0.0001), and MAC (r = 0.116, *p* < 0.0001), while negatively correlated with LVEF (r = −0.215, *p* < 0.0001). Additionally, regurgitant volume showed positive correlations with hemodynamic parameters, including systolic pulmonary artery pressure (r = 0.281, *p* < 0.0001) and tricuspid regurgitation (r = 0.190, *p* < 0.0001). Regarding laboratory findings, NT-proBNP levels were strongly correlated with regurgitant volume (r = 0.210, *p* < 0.0001) (Figure 3). These alterations indicate that cardiac remodeling occurs as FMR progresses, and the diastolic and systolic functions of the left ventricle gradually decline.

### 3.5. AFMR Independently Predicts All-Cause Mortality in CKM

**Figure 4:**
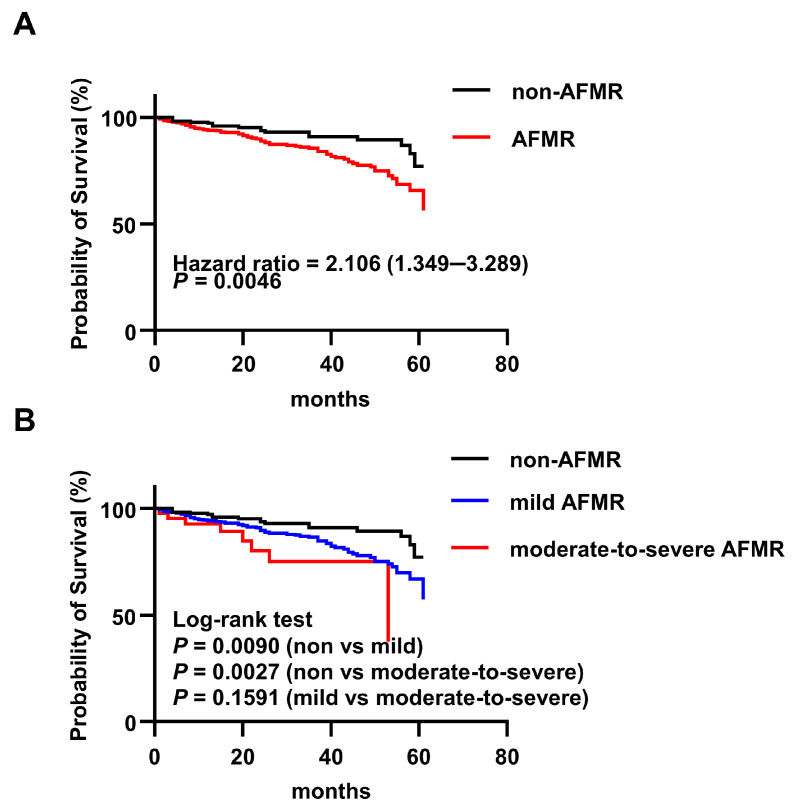
Kaplan–Meier analysis of all-cause mortality in patients with AFMR and CKM. (**A**) Kaplan–Meier survival curves were generated for patients with CKM syndrome with (*n* = 406) or without (*n* = 170) atrial functional mitral regurgitation (AFMR). (**B**) Kaplan–Meier survival curves were generated for patients with CKM syndrome among non-AFMR (*n* = 170), mild AFMR (*n* = 363) and moderate–severe AFMR (*n* = 43).

Among 576 patients assessed for atrial functional mitral regurgitation (AFMR), 406 (70.5%) were positive (AFMR group) and 170 were negative (non-AFMR group). The clinical features of the patients are shown in Table 4 and Table A3. The mean age was 55.18 ± 14.45 years, and 310 (53.82%) were male. During the median follow-up period of 36 months, 86 all-cause deaths occurred (non-AFMR, 17 [10%]; AFMR, 69 [17%]). Among the 86 deceased patients who underwent AFMR assessment, 23 were excluded owing to unknown causes of death. Among the remaining 63 patients, cardiovascular mortality was 50.0% (7/14) in the non-AFMR group, 88.1% (37/42) in the mild AFMR group, and 100% (7/7) in the moderate-to-severe AFMR group, suggesting a similar trend of increasing cardiovascular death with AFMR severity. Kaplan–Meier analysis indicated a significant association between AFMR and mortality, with a 5.95-year survival rate of 90% for non-AFMR patients and 83% for AFMR patients (HR, 2.106; 95% CI, 1.349–3.289; *p* = 0.0046) (Figure 4A). Univariate Cox regression analysis identified age, CHD, prior MI, AF, stroke, PH, red blood cell count, calcium level, and AFMR as significant determinants. After multivariable adjustment for these factors, AFMR remained an independent predictor of all-cause mortality (HR, 1.87; 95% CI, 1.08–3.24; *p* = 0.025) (Table 5). The proportional hazards assumption was also met for AFMR (*p* = 0.885). The C-index for the AFMR model was 0.700 (95% CI, 0.632–0.768), indicating acceptable predictive performance.

### 3.6. Association Between AFMR Severity and Mortality Risk

When stratified by severity (Table 6 and Table A4), patients with moderate-to-severe AFMR had a significantly higher prevalence of AF and PH than both the non-AFMR and mild AFMR groups. They also exhibited larger LAD, higher E/e’, and greater tricuspid regurgitation, indicating more advanced left atrial enlargement and diastolic dysfunction. Survival analysis demonstrated a graded increase in mortality risk with increasing AFMR severity. Compared to the non-AFMR group, both mild AFMR (5.95-year survival, 83.2% vs. 90%; HR, 2.009; 95% CI, 1.265–3.191; *p* = 0.0090) and moderate-to-severe AFMR (5.95-year survival, 81.4% vs. 90%; HR: 3.294, 95% CI, 1.007–10.78; *p* = 0.0027) were associated with significantly higher risk. The hazard ratio for moderate-to-severe versus mild AFMR was elevated but did not reach statistical significance (HR, 1.681; 95% CI, 0.676–4.180; *p* = 0.1591) (Figure 4B).

## 4. Discussion

In this retrospective cohort study of 1201 patients with CKM syndrome, we provide comprehensive data on the prevalence, associated risk profiles, and prognostic implications of FMR and AFMR. Our principal findings are threefold. First, FMR was identified as a significant independent prognostic factor, associated with elevated all-cause mortality in this high-risk population, thereby highlighting its adverse prognostic burden in CKM syndrome. Second, our analysis of echocardiographic parameters revealed a graded relationship between FMR severity and worsening left ventricular diastolic dysfunction. Finally, we demonstrated that AFMR was associated with increased all-cause mortality.

Although the pathophysiological mechanisms and clinical impact of FMR have been extensively studied, prior research has predominantly focused on its association with HF and T2DM, particularly in the context of HF with reduced ejection fraction (HFrEF) [17,18]. In contrast, data regarding the epidemiological characteristics and prognostic significance of the FMR within the high-risk CKM population remain scarce, especially in patients with ESRD, where concomitant cardiac, renal, and metabolic dysfunctions interact. In our cohort, patients with FMR exhibited larger cardiac chamber dimensions and higher NT-proBNP levels, indicating more advanced structural remodeling and hemodynamic stress. Therefore, consistent with the current mechanistic understanding, FMR may serve as a marker of disease severity. At the same time, regurgitant volume can exacerbate ventricular loading conditions and promote progressive chamber dilation, creating a vicious cycle of remodeling and progression of HF. Thus, FMR likely represents both a marker of advanced cardiometabolic disease and a potential contributor to the worsening of clinical outcomes.

FMR is common in patients who have HFpEF and is associated with increased symptom burden, hospitalization rates, and mortality [19,20]. Its presence in patients with HFpEF often signifies more pronounced left ventricular systolic dysfunction, left atrial myopathy, and exacerbated pulmonary vascular disease, all of which are strongly correlated with mortality risk [21,22]. While some studies suggest that only moderate or worse FMR confers an increased mortality risk [23], others report that even mild FMR elevates the risk of composite endpoints (e.g., death or HF hospitalization) [11]. Our findings extend this discourse to CKM syndrome, indicating that even mild FMR is associated with an increased risk of all-cause mortality. Notably, the prevalence of FMR in our CKM cohort (79.7%) was substantially higher than that reported in patients with HFpEF (41.8%) [22] and in those following non-ST-segment elevation acute coronary syndrome (40.1%) [24,25], highlighting the pronounced vulnerability of patients with CKM syndrome.

The interplay between FMR and metabolic disease has also been illustrated in patients with T2DM. FMR progression in T2DM is associated with adverse left ventricular remodeling, diastolic dysfunction, and compromised left atrial compliance, which contribute to higher mortality [26,27,28]. A study of patients with T2DM with morphologically normal mitral valves reported a 32% prevalence of mitral regurgitation, with even mild regurgitation linked to higher all-cause mortality [13], aligning with our observations. The remarkably higher prevalence of FMR (79.7%) in our CKM cohort likely reflects the synergistic cardiorenal–metabolic injury inherent in this syndrome.

FMR can generally be categorized into two main types: ventricular and atrial, which often coexist. Approximately two thirds of cases are attributable to left ventricular remodeling, commonly seen as reduced ejection fraction (HFrEF) or localized adverse remodeling with papillary muscle displacement; less frequently, ischemic papillary muscle dysfunction contributes. The remaining one third primarily involves atrial and annular dilation, often due to atrial fibrillation/flutter or chronically elevated left atrial pressure from diastolic dysfunction, particularly in patients with HFpEF [29,30]. AFMR, which is increasingly recognized in HFpEF, is associated with poorer outcomes. A recent study revealed that 35% of 429 patients with HFpEF exhibited AFMR, with 11% demonstrating moderate or worse severity [31]. In our CKM cohort, AFMR was highly prevalent (70.5%), with 10.6% of cases being moderate-to-severe AFMR. One study indicated that AFMR patients with HFpEF experienced a greater symptom burden and a mortality rate of 18.5%, in contrast to AFMR patients without HFpEF [32], whose mortality rate was comparable to the 17% observed in AFMR patients with CKM syndrome. While another study involving 189 patients with HFpEF or HFrEF found no significant mortality difference between the non-AFMR and AFMR groups [33], our analysis indicates that even mild AFMR increases the all-cause mortality risk in patients with CKM. This discrepancy may be attributed to differences in cohort characteristics, comorbidities, and sample sizes.

The mechanisms linking FMR to adverse outcomes in CKM are multifactorial, involving pressure–volume overload and syndrome-specific pathophysiological interactions. In HF, FMR typically results from altered mitral leaflet tethering forces due to ventricular or atrial remodeling, leading to elevated left ventricular filling pressures, pulmonary venous hypertension, and left atrial dilation, all prognostic markers [34]. Additional factors exacerbate this cascade in CKM. Diabetes promotes left atrial remodeling, while volume overload from FMR may worsen the pressure–volume relationship in a ventricle that is often already compromised by diastolic dysfunction. ESRD frequently induces left ventricular hypertrophy (present in approximately 50% of patients) [35], further altering cardiac mechanics. Consistent with this, our CKM patients with FMR exhibited higher rates of PH, left atrial dilation, left ventricular hypertrophy, and elevated E/e’ ratio—all indices of worsening diastolic dysfunction that correlated with regurgitation severity. A similar trend is observed in the AFMR subgroup. We also observed a significantly higher prevalence of systolic pulmonary artery pressure, MAC, and tricuspid regurgitation (TR) in the FMR and AFMR group. PH in HFpEF, which is often associated with FMR, independently predicts mortality [36,37], a finding that was echoed in our cohort. MAC, reported in 25–59% of dialysis patients [38], was present in 49.11% and 41.87% of our FMR and AFMR groups, respectively, with severity escalating alongside the regurgitation grade. Given its established link to cardiovascular mortality, MAC l is likely to contribute to poor prognosis. Furthermore, TR, prevalent in 30–50% of patients with chronic severe MR [39] and exacerbated by CKD [40], was found in 79.02% of our CKM cohort, reflecting advanced cardiorenal compromise.

CKM syndrome entails a multidirectional pathophysiology in which cardiac, renal, and metabolic dysfunctions synergistically increase morbidity and mortality beyond the sum of the individual components [6]. CKD, especially concomitant diabetes, creates a proinflammatory state that increases cardiovascular risk [41]. CKD-induced anemia and mineral bone disease aggravate cardiovascular injury [42]. Conversely, HF reduces renal perfusion via low cardiac output, high venous pressure, and neurohormonal activation, worsening renal function and fluid retention, resulting in a vicious cycle of cardiorenal deterioration [43]. In our study, echocardiographic markers of diastolic dysfunction (E/e’, LAD, and LVIDd) worsened, LVEF declined, and NT-proBNP levels increased with increasing FMR/AFMR severity. This suggests that FMR/AFMR exacerbates underlying diastolic dysfunction in CKM, potentially precipitating systolic impairment over time.

The therapeutic management of FMR is determined by its underlying pathophysiology and disease severity [44]. According to the 2025 update of the European Society of Cardiology/European Association for Cardio-Thoracic Surgery Valvular Heart Disease Guidelines, guideline-directed medical therapy remains the foundation of treatment, whereas transcatheter edge-to-edge repair is recommended for selected symptomatic patients with impaired left ventricular ejection fraction (<50%) and persistent severe ventricular FMR despite optimized medical therapy and cardiac resynchronization therapy [45]. However, the long-term efficacy of valve repair may be limited in advanced or ongoing ventricular remodeling, which remains a major challenge in the management of secondary mitral regurgitation [46,47]. Careful patient selection is critical. Patients whose clinical status is primarily driven by severe FMR rather than global ventricular remodeling, particularly those with disproportionate FMR, are more likely to derive meaningful benefits from valvular interventions.

This study has some limitations that should be considered when interpreting the results. First, its retrospective, single-center design and relatively modest sample size may have introduced selection bias and limited the generalizability of the findings. The short follow-up duration further restricted the ability to assess long-term outcomes. Although we adjusted for known confounders, residual confounders may have persisted owing to the observational nature of the study. Additionally, the study cohort was limited to patients with HFpEF. Finally, whether therapies that reduce FMR severity in the general population can improve outcomes, specifically in patients with CKM, requires future investigation in prospective studies.

## 5. Conclusions

FMR and AFMR are highly prevalent and prognostically significant in CKM syndrome, with even mild degrees associated with increased all-cause mortality. The underlying mechanism appears to involve the aggravation of pre-existing cardiac structural and functional abnormalities—particularly diastolic dysfunction—within the complex cardiorenal–metabolic milieu of CKM, thereby accelerating the trajectory towards pump failure and death. These findings advocate for the systematic echocardiographic assessment of mitral regurgitation in patients with CKM and underscore the need for future research on targeted management strategies to mitigate the prognostic burden imposed by FMR in this high-risk population.

## Figures and Tables

**Table 1:** Clinical characteristics of non-FMR and FMR patients in the cohort.

	Total (*n* = 1201)	Non-FMR (*n* = 244)	FMR (*n* = 957)	*p* Value
Demographic data				
Male, n (%)	695 (57.87%)	141 (57.79%)	554 (57.89%)	0.977
Age, (year)	56.58 (13.94)	54.76 (13.56)	57.05 (14.00)	0.022 *
Any comorbidity				
Coronary heart disease, *n* (%)	569 (47.50%)	97 (39.92%)	472 (49.42%)	0.008 *
Prior myocardial infarction, *n* (%)	39 (3.25%)	7 (2.87%)	32 (3.34%)	0.709
Hypertension, *n* (%)	1123 (93.51%)	227 (93.03%)	896 (93.63%)	0.737
Diabetes, *n* (%)	479 (39.92%)	73 (29.92%)	406 (42.47%)	<0.001 *
AF, *n* (%)	40 (3.33%)	1 (0.41%)	39 (4.08%)	0.004 *
Prior stroke, *n* (%)	218 (18.15%)	38 (15.57%)	180 (18.81%)	0.242
PH, *n* (%)	184 (15.32%)	9 (3.69%)	175 (18.29%)	<0.001 *
Echocardiographic parameters				
LAD, (mm)	41.65 (5.61)	38.2 (6.01)	42.53 (5.15)	<0.001 *
LVIDd, (mm)	49.79 (5.43)	47.52 (5.24)	50.36 (5.33)	<0.001 *
LVPW, (mm)	10.75 (1.89)	10.34 (1.85)	10.86 (1.89)	<0.001 *
IVST, (mm)	10.84 (1.95)	10.45 (1.94)	10.94 (1.94)	<0.001 *
E/e’	15.51 (5.96)	12.84 (4.23)	16.20 (6.15)	<0.001 *
LVEF, (%)	59.82 (4.60)	61.58 (4.70)	59.37 (4.46)	<0.001 *
MAC, *n* (%)	565 (47.04%)	95 (38.93%)	470 (49.11%)	0.004 *
Tricuspid regurgitation, *n* (%)	949 (79.02%)	127 (52.05%)	822 (85.89%)	<0.001 *
Regurgitant volume, (mL)	13.31 (14.59)	0.59 (4.24)	16.58 (14.52)	<0.001 *
Laboratory examination				
NT-proBNP (pg/mL)	12,751.09 (12,545.81)	7548.06 (10,235.12)	13,924.24 (12,726.91)	<0.001 *
Creatinine (μmol/L)	871.63 (511.24)	868.26 (340.83)	872.48 (546.27)	0.909
Urea (mmol/L)	31.35 (97.71)	27.74 (11.21)	32.26 (109.27)	0.523
Blood glucose (mmol/L)	6.38 (3.84)	6.30 (3.80)	6.40 (3.85)	0.708
TC (mmol/L)	4.11 (1.33)	4.18 (1.22)	4.10 (1.36)	0.393
TG (mmol/L)	1.67 (0.98)	1.83 (1.08)	1.63 (0.95)	0.011 *
HDL (mmol/L)	0.94 (0.34)	0.92 (0.34)	0.95 (0.34)	0.311
LDL (mmol/L)	2.56 (1.05)	2.59 (0.98)	2.55 (1.07)	0.617
v-LDL (mmol/L)	0.61 (0.35)	0.66 (0.37)	0.59 (0.34)	0.005 *

Continuous variables are presented as means (standard deviation, SD), and categorical variables are presented as counts (%). *t* test and chi-square test are used to compare the difference between non-FMR and FMR patients. *, *p* < 0.05. Abbreviations: AF, atrial fibrillation; LAD, left atrial diameter; LVIDd, left ventricular internal dimension at end-diastole; LVPW, left ventricular posterior wall thickness; IVST, interventricular septal thickness; E/e’, ratio of early mitral inflow velocity (E) to early diastolic mitral annular velocity (e’); LVEF, left ventricular ejection fraction; MAC, mitral annular calcification; NT-proBNP, N-terminal pro-B-type Natriuretic Peptide; TC, total cholesterol; TG, triglycerides; HDL, high-density lipoprotein cholesterol; LDL, low-density lipoprotein cholesterol; v-LDL, very-low-density lipoprotein cholesterol; PH, pulmonary hypertension; FMR, functional mitral regurgitation.

**Table 2:** Univariable and multivariable Cox regression analysis of risk factors for all-cause mortality in the FMR cohort.

Variable	Univariable		Multivariable	
	*p* Value	HR [95% CI]	*p* Value	HR [95% CI]
Sex	0.853	0.97 [0.73, 1.30]	Not selected	
Age	0.000	1.05 [1.04, 1.06]	<0.001	1.04 [1.02, 1.05]
Coronary heart disease	0.000	2.28 [1.69, 3.09]	0.004	1.60 [1.16, 2.19]
Prior myocardial infarction	0.015	2.21 [1.17, 4.18]	0.247	1.48 [0.76, 2.86]
Hypertension	0.897	1.04 [0.58, 1.87]	Not selected	
Diabetes	0.019	1.41 [1.06, 1.89]	0.632	1.08 [0.78, 1.51]
AF	0.001	2.69 [1.50, 4.84]	0.108	1.65 [0.90, 3.05]
Prior stroke	0.002	1.69 [1.20, 2.37]	0.093	1.35 [0.95, 1.90]
PH	0.103	1.37 [0.94, 2.00]	Not selected	
ARNI	0.464	1.12 [0.83, 1.52]	Not selected	
ACEI/ARB	0.256	1.25 [0.85, 1.83]	Not selected	
β-blocker	0.671	0.93 [0.66, 1.31]	Not selected	
CCB	0.400	1.13 [0.85, 1.51]	Not selected	
Creatinine	0.001	1.00 [1.00, 1.00]	0.414	1.00 [1.00, 1.00]
Urea	0.193	0.99 [0.98, 1.00]	Not selected	
WBC	0.449	1.01 [0.98, 1.05]	Not selected	
NEU	0.471	1.01 [0.98, 1.06]	Not selected	
LYM	0.745	0.96 [0.75, 1.22]	Not selected	
RBC	0.005	1.33 [1.09, 1.62]	0.070	1.22 [0.98, 1.52]
Hemoglobin	0.408	1.00 [1.00, 1.01]	Not selected	
Platelet	0.084	1.00 [1.00, 1.00]	0.273	1.00 [1.00, 1.00]
Calcium	0.021	1.60 [1.07, 2.39]	0.250	1.31 [0.83, 2.06]
Phosphorus	0.365	0.91 [0.74, 1.11]	Not selected	
Blood glucose	0.042	1.03 [1.00, 1.06]	0.412	1.02 [0.98, 1.05]
FMR	0.002	1.85 [1.25, 2.73]	0.019	1.64 [1.09, 2.48]

Abbreviations: AF, atrial fibrillation; ACEI/ARB, angiotensin converting enzyme inhibitors/angiotonin receptor blocker; CCB, calcium channel blockers; PH, pulmonary hypertension; WBC, white blood cell; NEU, neutrophils; LYM, lymphocyte; RBC, red blood cell; FMR, functional mitral regurgitation. Multivariable adjusted for: age; coronary heart disease; prior myocardial infarction; diabetes; AF; prior stroke; creatinine; RBC; platelet; calcium; blood glucose.

**Table 3:** Clinical characteristics of non-FMR, mild FMR and moderate-to-severe FMR patients in the cohort.

	Total (*n* = 1201)	Non-FMR (*n* = 244)	Mild FMR (*n* = 801)	Moderate-to-Severe FMR (*n* = 156)
Demographic data				
Male, n (%)	695 (57.87%)	141 (57.79%)	468 (58.43%)	86 (55.13%)
Age, (year)	56.58 (13.94)	54.76 (13.56)	56.54 (13.82)	59.64 (14.68) #,†
Any comorbidity				
Coronary heart disease, n (%)	569 (47.50%)	97 (39.92%)	393 (49.19%) *	79 (50.64%) #
Prior myocardial infarction, n (%)	39 (3.25%)	7 (2.87%)	25 (3.12%)	7 (4.49%)
Hypertension, n (%)	1123 (93.51%)	227 (93.03%)	744 (92.88%)	152 (97.44%)
Diabetes, n (%)	479 (39.92%)	73 (29.92%)	343 (42.88%) *	63 (40.38%) #
AF, n (%)	40 (3.33%)	1 (0.41%)	26 (3.25%) *	13 (8.33%) #,†
Prior stroke, n (%)	218 (18.15%)	38 (15.57%)	147 (18.35%)	33 (21.15%)
PH, n (%)	184 (15.32%)	9 (3.69%)	115 (14.36%) *	60 (38.46%) #,†
Echocardiographic parameters				
LAD, (mm)	41.65 (5.61)	38.2 (6.01)	41.98 (4.77) *	45.34 (6.08) #,†
LVIDd, (mm)	49.79 (5.43)	47.52 (5.24)	49.92 (5.15) *	52.64 (5.66) #,†
LVPW, (mm)	10.75 (1.89)	10.34 (1.85)	10.86 (1.89) *	10.89 (1.89) #
IVST, (mm)	10.84 (1.95)	10.45 (1.94)	10.92 (1.96) *	11.02 (1.84) #
E/e’	15.51 (5.96)	12.84 (4.23)	15.51 (5.61) *	19.83 (7.42) #,†
LVEF, (%)	59.82 (4.60)	61.58 (4.70)	59.75 (4.39) *	57.42 (4.34) #,†
MAC, *n* (%)	565 (47.04%)	95 (38.93%)	377 (47.07%) *	93 (59.62%) #,†
Tricuspid regurgitation, *n* (%)	949 (79.02%)	127 (52.05%)	671 (83.77%) *	151 (96.79%) #,†
Regurgitant volume, (mL)	13.31 (14.59)	0.59(4.24)	12.1(8.87) *	39.74(15.98) #,†
Laboratory examination				
NT-proBNP (pg/mL)	12,751.09 (12,545.81)	7548.06 (10,235.12)	12,549.85 (12,063.92) *	20,083.04 (13,815.82) #,†
Creatinine (μmol/L)	871.63 (511.24)	868.26 (340.83)	869.48 (482.01)	887.71 (798.06)
Urea (mmol/L)	31.35 (97.71)	27.74 (11.21)	33.43 (119.46)	26.33 (10.81)
Blood glucose (mmol/L)	6.38 (3.84)	6.30 (3.80)	6.47 (3.99)	6.04 (3.04)
TC (mmol/L)	4.11 (1.33)	4.18 (1.22)	4.10 (1.34)	4.07 (1.46)
TG (mmol/L)	1.67 (0.98)	1.83 (1.08)	1.68 (0.97)	1.38 (0.80) #,†
HDL (mmol/L)	0.94 (0.34)	0.92 (0.34)	0.94 (0.33)	1 (0.36)
LDL (mmol/L)	2.56 (1.05)	2.59 (0.98)	2.56 (1.06)	2.52 (1.10)
v-LDL (mmol/L)	0.61 (0.35)	0.66 (0.37)	0.6 (0.33) *	0.56 (0.37) #

Continuous variables are presented as means (SD), and categorical variables are presented as counts (%). Chi-square test and one-way ANOVA test are used to compare the difference among non-FMR, mild FMR and moderate-to-severe FMR patients. non-FMR versus mild FMR, * *p* < 0.05; non-FMR versus moderate-to-severe FMR, # *p* < 0.05; mild FMR versus moderate-to-severe FMR, † *p* < 0.05. Abbreviations: AF, atrial fibrillation; LAD, left atrial diameter; LVIDd, left ventricular internal dimension at end-diastole; LVPW, left ventricular posterior wall thickness; IVST, interventricular septal thickness; E/e’, ratio of early mitral inflow velocity (E) to early diastolic mitral annular velocity (e’); LVEF, left ventricular ejection fraction; MAC, mitral annular calcification; NT-proBNP, N-terminal pro-B-type Natriuretic Peptide; TC, total cholesterol; TG, triglycerides; HDL, high-density lipoprotein cholesterol; LDL, low-density lipoprotein cholesterol; v-LDL, very-low-density lipoprotein cholesterol; PH, pulmonary hypertension; FMR, functional mitral regurgitation.

**Table 4:** Clinical characteristics of non-AFMR and AFMR patients in the cohort.

	Total (*n* = 576)	Non-AFMR (*n* = 170)	AFMR (*n* = 406)	*p* Value
Demographic data				
Male, *n* (%)	310 (53.82%)	92 (54.12%)	218 (53.69%)	0.926
Age, (year)	55.18 (14.45)	53.45 (13.89)	55.91 (14.64)	0.062
Any comorbidity				
Coronary heart disease, *n* (%)	235 (40.87%)	61 (36.09%)	174 (42.86%)	0.133
Prior myocardial infarction, *n* (%)	12 (2.08%)	3 (1.76%)	9 (2.22%)	0.979
Hypertension, *n* (%)	535 (92.88%)	156 (91.76%)	379 (93.35%)	0.500
Diabetes, *n* (%)	217 (37.67%)	49 (28.82%)	168 (41.38%)	0.005 *
AF, *n* (%)	16 (2.78%)	1 (0.59%)	15 (3.69%)	0.073
Prior stroke, *n* (%)	91 (15.80%)	24 (14.12%)	67 (16.50%)	0.474
PH, *n* (%)	71 (12.33%)	7 (4.12%)	64 (15.76%)	<0.001 *
Echocardiographic parameters				
LAD, (mm)	39.9 (5.42)	37.33 (5.70)	40.98 (4.92)	<0.001 *
LVIDd, (mm)	47.63 (4.19)	46.42 (4.20)	48.13 (4.08)	<0.001 *
E/e’	14.42 (5.52)	12.7 (4.47)	15.15 (5.76)	<0.001 *
LVPW, (mm)	10.5 (1.86)	10.17 (1.90)	10.64 (1.83)	0.006 *
IVST, (mm)	10.52 (1.89)	10.23 (1.96)	10.64 (1.85)	0.0180 *
E/e’	14.42 (5.52)	12.7 (4.47)	15.15 (5.76)	<0.001 *
LVEF, (%)	61.54 (4.28)	62.42 (4.39)	61.17 (4.18)	0.001 *
MAC, *n* (%)	230 (39.93%)	60 (35.29%)	170 (41.87%)	0.142
Tricuspid regurgitation, *n* (%)	413 (71.7%)	87 (51.18%)	326 (80.30%)	<0.001 *
Regurgitant volume, (mL)	9.85 (12.16)	0.55 (3.84)	13.77 (12.33)	<0.001 *
Laboratory examination				
NT-proBNP (pg/mL)	10,439.31 (11,996.59)	7062.53 (9953.49)	11,663.39 (12,449.98)	0.001 *
Creatinine (μmol/L)	873.45 (526.37)	874.11 (350.51)	873.18 (584.32)	0.985
Urea (mmol/L)	27.5 (12.06)	27.69 (11.36)	27.42 (12.36)	0.811
Blood glucose (mmol/L)	6.38 (4.37)	6.31 (4.03)	6.40 (4.51)	0.822
TC (mmol/L)	4.25 (1.35)	4.28 (1.22)	4.23 (1.41)	0.690
TG (mmol/L)	1.73 (0.97)	1.90 (1.04)	1.66 (0.93)	0.006 *
HDL (mmol/L)	0.95 (0.35)	0.92 (0.33)	0.97 (0.35)	0.150
LDL (mmol/L)	2.66 (1.07)	2.69 (1.00)	2.64 (1.10)	0.664
v-LDL (mmol/L)	0.64 (0.34)	0.68 (0.36)	0.62 (0.34)	0.064

Continuous variables are presented as means (SD), and categorical variables are presented as counts (%). *t* test and chi-square test are used to compare the difference between non-AFMR and AFMR patients. *, *p* < 0.05. Abbreviations: AF, atrial fibrillation; LAD, left atrial diameter; LVIDd, left ventricular internal dimension at end-diastole; LVPW, left ventricular posterior wall thickness; IVST, interventricular septal thickness; E/e’, ratio of early mitral inflow velocity (E) to early diastolic mitral annular velocity (e’); LVEF, left ventricular ejection fraction; MAC, mitral annular calcification; NT-proBNP, N-terminal pro-B-type Natriuretic Peptide; TC, total cholesterol; TG, triglycerides; HDL, high-density lipoprotein cholesterol; LDL, low-density lipoprotein cholesterol; v-LDL, very-low-density lipoprotein cholesterol; PH, pulmonary hypertension; AFMR, atrial functional mitral regurgitation.

**Table 5:** Univariable and multivariable Cox regression analysis of risk factors for all-cause mortality in the AFMR cohort.

Variable	Univariable		Multivariable	
	*p* Value	HR [95%CI]	*p* Value	HR [95%CI]
Sex	0.946	0.99 [0.64, 1.51]	Not selected	
Age	0.000	1.04 [1.02, 1.06]	0.003	1.03 [1.01, 1.05]
Coronary heart disease	0.000	2.24 [1.46, 3.45]	0.047	1.59 [1.01, 2.52]
Prior myocardial infarction	0.063	3.00 [0.94, 9.54]	0.254	1.99 [0.61, 6.48]
Hypertension	0.881	0.94 [0.43, 2.04]	Not selected	
Diabetes	0.115	1.41 [0.92, 2.15]	Not selected	
AF	0.003	3.59 [1.56, 8.26]	0.233	1.74 [0.70, 4.36]
Prior stroke	0.061	1.66 [0.98, 2.84]	0.255	1.37 [0.80, 2.37]
PH	0.021	1.94 [1.11, 3.40]	0.530	1.21 [0.66, 2.22]
ARNI	0.626	0.88 [0.54, 1.46]	Not selected	
ACEI/ARB	0.145	1.50 [0.87, 2.58]	Not selected	
β-blocker	0.705	0.90 [0.53, 1.54]	Not selected	
CCB	0.830	1.05 [0.68, 1.60]	Not selected	
Creatinine	0.101	1.00 [1.00, 1.00]	Not selected	
Urea	0.931	1.00 [0.98, 1.02]	Not selected	
WBC	0.570	1.01 [0.96, 1.07]	Not selected	
NEU	0.531	1.02 [0.96, 1.07]	Not selected	
LYM	0.956	0.99 [0.70, 1.40]	Not selected	
RBC	0.001	1.61 [1.21, 2.15]	0.018	1.46 [1.07, 2.00]
Hemoglobin	0.121	1.01 [1.00, 1.02]	Not selected	
Platelet	0.489	1.00 [1.00, 1.00]	Not selected	
Calcium	0.043	1.77 [1.02, 3.07]	0.631	1.16 [0.63, 2.15]
Phosphorus	0.804	0.96 [0.72, 1.29]	Not selected	
Blood glucose	0.205	1.03 [0.99, 1.07]	Not selected	
AFMR	0.006	2.12 [1.25, 3.61]	0.025	1.87 [1.08, 3.24]

Abbreviations: AF, atrial fibrillation; ACEI/ARB, angiotensin converting enzyme inhibitors/angiotonin receptor blocker; CCB, calcium channel blockers; PH, pulmonary hypertension; WBC, white blood cell; NEU, neutrophils; LYM, lymphocyte; RBC, red blood cell; AFMR, atrial functional mitral regurgitation. Multivariable adjusted for: age; coronary heart disease; prior myocardial infarction; hyperlipemia; AF; prior stroke; PH; RBC; calcium.

**Table 6:** Clinical characteristics of non-AFMR, mild AFMR and moderate-to-severe AFMR patients in the cohort.

	Total (*n* = 576)	Non-AFMR (*n* = 170)	Mild AFMR (*n* = 363)	Moderate-to-Severe AFMR (*n* = 43)
Demographic data				
Male, *n* (%)	310 (53.82%)	92 (54.12%)	196 (53.99%)	22 (51.16%)
Age, (year)	55.18 (14.45)	53.45 (13.89)	55.66 (14.42)	58.07 (16.43)
Any comorbidity				
Coronary heart disease, *n* (%)	235 (40.87%)	61 (36.09%)	155 (42.7%)	19 (44.19%)
Prior myocardial infarction, *n* (%)	12 (2.08%)	3 (1.76%)	8 (2.20%)	1 (2.33%)
Hypertension, *n* (%)	535 (92.88%)	156 (91.76%)	336 (92.56%)	43 (100.00%)
Diabetes, *n* (%)	217 (37.67%)	49 (28.82%)	147 (40.50%) *	21 (48.84%) #
AF, *n* (%)	16 (2.78%)	1 (0.59%)	8 (2.20%)	7 (16.28%) #,†
Prior stroke, *n* (%)	91 (15.80%)	24 (14.12%)	56 (15.43%)	11 (25.58%)
PH, *n* (%)	71 (12.33%)	7 (4.12%)	48 (13.22%) *	16 (37.21%) #,†
Echocardiographic parameters				
LAD, (mm)	39.9 (5.42)	37.33 (5.70)	40.65 (4.45) *	43.83 (7.31) #,†
LVIDd, (mm)	47.63 (4.19)	46.42 (4.20)	48.1 (4.12) *	48.46 (3.79) #
LVPW, (mm)	10.5 (1.86)	10.17 (1.90)	10.63 (1.83) *	10.71 (1.80)
IVST, (mm)	10.52 (1.89)	10.23 (1.96)	10.63 (1.88)	10.73 (1.62)
E/e’	14.42 (5.52)	12.7 (4.47)	14.66 (5.21) *	19.23 (8.17) #,†
LVEF, (%)	61.54 (4.28)	62.42 (4.39)	61.39 (4.13) *	59.3 (4.16) #,†
MAC, *n* (%)	230 (39.93%)	60 (35.29%)	148 (40.77%)	22 (51.16%)
Tricuspid regurgitation, *n* (%)	413 (71.70%)	87 (51.18%)	284 (78.24%) *	42 (97.67%) #,†
Regurgitant volume, (mL)	9.85 (12.16)	0.55 (3.84)	10.88 (8.11) *	38.02 (14.87) #,†
Laboratory examination				
NT-proBNP (pg/mL)	10,439.31 (11,996.59)	7062.53 (9953.49)	10,732.89 (11,323.35) *	18,433.6 (17,533.33) #
Creatinine (μmol/L)	873.45 (526.37)	874.11 (350.51)	852.73 (381.29)	1044.43 (1411.29)
Urea (mmol/L)	27.5 (12.06)	27.69 (11.36)	27.54 (12.43)	26.49 (11.80)
Blood glucose (mmol/L)	6.38 (4.37)	6.31 (4.03)	6.43 (4.59)	6.18 (3.81)
TC (mmol/L)	4.25 (1.35)	4.28 (1.22)	4.2 (1.41)	4.47 (1.36)
TG (mmol/L)	1.73 (0.97))	1.9 (1.04)	1.67 (0.96) *	1.54 (0.69) #
HDL (mmol/L)	0.95 (0.35)	0.92 (0.33)	0.95 (0.34)	1.12 (0.43) #
LDL (mmol/L)	2.66 (1.07)	2.69 (1.00)	2.63 (1.10)	2.77 (1.11)
v-LDL (mmol/L)	0.64 (0.34)	0.68 (0.36)	0.63 (0.34)	0.59 (0.28)

Continuous variables are presented as means (SD), and categorical variables are presented as counts (%). Chi-square test and one-way ANOVA test are used to compare the difference among non-AFMR, mild AFMR and moderate-to-severe AFMR patients. non-AFMR versus mild AFMR, * *p* < 0.05; non-AFMR versus moderate-to-severe AFMR, # *p* < 0.05; mild AFMR versus moderate-to-severe AFMR, † *p* < 0.05. Abbreviations: AF, atrial fibrillation; LAD, left atrial diameter; LVIDd, left ventricular internal dimension at end-diastole; LVPW, left ventricular posterior wall thickness; IVST, interventricular septal thickness; E/e’, ratio of early mitral inflow velocity (E) to early diastolic mitral annular velocity (e’); LVEF, left ventricular ejection fraction; MAC, mitral annular calcification; NT-proBNP, N-terminal pro-B-type Natriuretic Peptide; TC, total cholesterol; TG, triglycerides; HDL, high-density lipoprotein cholesterol; LDL, low-density lipoprotein cholesterol; v-LDL, very-low-density lipoprotein cholesterol; PH, pulmonary hypertension; AFMR, atrial functional mitral regurgitation.

## Data Availability

The data presented in this study are available on request from the corresponding authors.

## Abbreviations

The following abbreviations are used in this manuscript:

AHA	American Heart Association
AF	Atrial Fibrillation
AFMR	Atrial Functional Mitral Regurgitation
CI	Confidence Interval
CKD	Chronic Kidney Disease
CKM	Cardiovascular–Kidney–Metabolic
EF	Ejection Fraction
ESRD	End-Stage Renal Disease
FMR	Functional Mitral Regurgitation
HF	Heart Failure
HFpEF	Heart Failure with preserved Ejection Fraction
HFrEF	Heart Failure with reduced Ejection Fraction
HR	Hazard Ratio
IQR	Interquartile Range
IVST	Interventricular Septal Thickness
LA	Left Atrium/Left Atrial
LAD	Left Atrium Diameter
LV	Left Ventricular
LVIDd	Left Ventricular Internal Diameter at end-diastole
LVEF	Left Ventricular Ejection Fraction
LVPW	Left Ventricular Posterior Wall thickness
MAC	Mitral Annular Calcification
MR	Mitral Regurgitation
NPs	Natriuretic Peptides
NT-proBNP	N-terminal pro-B-type Natriuretic Peptide
NSTSEACS	non-ST-segment elevation acute coronary syndrome
PH	Pulmonary Hypertension
sPAP	systolic Pulmonary Artery Pressure
TTE	Transthoracic Echocardiography
VFMR	Ventricular Functional Mitral Regurgitation
v-LDL	very-low-density lipoprotein

## Appendix A

**Table A1:** Medication Use and Laboratory Parameters of non-FMR and FMR patients in the cohort.

	Total (n = 1201)	Non-FMR (n = 244)	FMR (n = 957)	*p* Value
Medications				
ARNI, n (%)	457 (38.05%)	66 (27.05%)	391 (40.86%)	<0.001 *
ACEI/ARB, n (%)	196 (16.32%)	28 (11.48%)	168 (17.55%)	0.022 *
β-blocker, n (%)	325 (27.06%)	45 (18.44%)	280 (29.26%)	0.001 *
CCB, n (%)	585 (48.71%)	95 (38.93%)	490 (51.20%)	0.001 *
Antidiabetic drug, n (%)	440 (37.80%)	64 (27.23%)	376 (40.47%)	<0.001 *
Lipid-lowering drug, n (%)	84 (6.99%)	10 (4.10%)	74 (7.73%)	0.147
Laboratory examination				
WBC (×10^9^/L)	7.14 (3.35)	11.38 (61.42)	7.06 (3.22)	0.273
NEU (×10^9^/L)	5.28 (3.15)	5.51 (3.63)	5.22 (3.01)	0.193
LYM (×10^9^/L)	1.13 (0.65)	1.21 (0.73)	1.11 (0.62)	0.034 *
RBC (×10^12^/L)	3.02 (0.73)	3.06 (0.77)	3.01 (0.72)	0.376
Hemoglobin (g/L)	87.54 (21.12)	89.88 (24.54)	86.95 (20.13)	0.086
Platelet (×10^9^/L)	199.49 (89.18)	207.16 (108.18)	197.53 (83.60)	0.196
Calcium (mmol/L)	2.05 (0.34)	2.06 (0.34)	2.05 (0.34)	0.519
Phosphorus (mmol/L)	2.11 (0.74)	2.17 (0.73)	2.09 (0.74)	0.149

Continuous variables are presented as means (standard deviation, SD), and categorical variables are presented as counts (%). *t* test and chi-square test are used to compare the difference between non-FMR and FMR patients. *, *p* < 0.05. Abbreviations: ACEI/ARB, angiotensin converting enzyme inhibitors/angiotonin receptor blocker; CCB, calcium channel blockers; WBC, white blood cell; NEU, neutrophils; LYM, lymphocyte; RBC, red blood cell; FMR, functional mitral regurgitation.

**Table A2:** Medication Use and Laboratory Parameters of non-FMR, mild FMR and moderate-to-severe FMR patients in the cohort.

	Total (*n* = 1201)	Non-FMR (*n* = 244)	Mild FMR (*n* = 801)	Moderate-to-Severe FMR (*n* = 156)
Medications				
ARNI, n (%)	457 (38.05%)	66 (27.05%)	318 (39.70%) *	73 (46.79%) #
ACEI/ARB, n (%)	196 (16.32%)	28 (11.48%)	134 (16.73%) *	34 (21.79%) #
β-blocker, n (%)	325 (27.06%)	45 (18.44%)	225 (28.09%) *	55 (35.26%) #
CCB, n (%)	585 (48.71%)	95 (38.93%)	393 (49.06%) *	97 (62.18%) #,†
Antidiabetic drug	440 (37.8%)	64 (27.23%)	318 (40.87%) *	58 (38.41%) #
Lipid-lowering drug	84 (6.99%)	10 (4.10%)	47 (5.86%)	27 (17.42%) #,†
Laboratory examination				
WBC (×10^9^/L)	7.14 (3.35)	11.38 (61.42)	7.09 (3.14)	6.86 (3.59)
NEU (×10^9^/L)	5.28 (3.15)	5.51 (3.63)	5.24 (2.93)	5.14 (3.40)
LYM (×10^9^/L)	1.13 (0.65)	1.21 (0.73)	1.13 (0.64)	1.03 (0.50) #
RBC (×10^12^/L)	3.02 (0.73)	3.06 (0.77)	3.01 (0.72)	3 (0.72)
Hemoglobin (g/L)	87.54 (21.12)	89.88 (24.54)	87.11 (20.08)	86.13 (20.41)
Platelet (×10^9^/L)	199.49 (89.18)	207.16 (108.18)	199.30 (84.52)	188.46 (78.34)
Calcium (mmol/L)	2.05 (0.34)	2.06 (0.34)	2.05 (0.34)	2.05 (0.34)
Phosphorus (mmol/L)	2.11 (0.74)	2.17 (0.73)	2.10 (0.75)	2.06 (0.70)

Continuous variables are presented as means (SD), and categorical variables are presented as counts (%). Chi-square test and one-way ANOVA test are used to compare the difference among non-FMR, mild FMR and moderate-to-severe FMR patients. non-FMR versus mild FMR, * *p* < 0.05; non-FMR versus moderate-to-severe FMR, # *p* < 0.05; mild FMR versus moderate-to-severe FMR, † *p* < 0.05. Abbreviations: ACEI/ARB, angiotensin converting enzyme inhibitors/angiotonin receptor blocker; CCB, calcium channel blockers; WBC, white blood cell; NEU, neutrophils; LYM, lymphocyte; RBC, red blood cell; FMR, functional mitral regurgitation.

**Table A3:** Medication Use and Laboratory Parameters of non-AFMR and AFMR patients in the cohort.

	Total (*n* = 576)	Non-AFMR (*n* = 170)	AFMR (*n* = 406)	*p* Value
Medications				
ARNI, *n* (%)	177 (30.73%)	42 (24.71%)	135 (33.25%)	0.043 *
ACEI/ARB, *n* (%)	88 (15.28%)	19 (11.18%)	69 (17.00%)	0.077
β-blocker, *n* (%)	141 (24.48%)	29 (17.06%)	112 (27.59%)	0.007 *
CCB, *n* (%)	248 (43.06%)	65 (38.24%)	183 (45.07%)	0.131
Antidiabetic drug, *n* (%)	199 (35.79%)	44 (26.67%)	155 (39.64%)	0.004
Lipid-lowering drug, *n* (%)	28 (4.86%)	5 (2.94%)	23 (5.67%)	0.166
Laboratory examination				
WBC (×10^9^/L)	7.26 (3.59)	7.50 (4.13)	7.16 (3.34)	0.313
NEU (×10^9^/L)	5.40 (3.42)	5.60 (3.95)	5.31 (3.18)	0.35
LYM (×10^9^/L)	1.16 (0.68)	1.18 (0.63)	1.15 (0.70)	0.692
RBC (×10^12^/L)	3.03 (0.73)	3.09 (0.77)	3.01 (0.71)	0.231
Hemoglobin (g/L)	88.44 (21.82)	90.48 (24.95)	87.59 (20.34)	0.147
Platelet (×10^9^/L)	198.91 (85.21)	204.49 (96.65)	196.57 (79.95)	0.310
Calcium (mmol/L)	2.06 (0.35)	2.07 (0.35)	2.06 (0.35)	0.693
Phosphorus (mmol/L)	2.11 (0.75)	2.19 (0.77)	2.07 (0.74)	0.102

Continuous variables are presented as means (SD), and categorical variables are presented as counts (%). *t* test and chi-square test are used to compare the difference between non-AFMR and AFMR patients. * *p* < 0.05. Abbreviations: ACEI/ARB, angiotensin converting enzyme inhibitors/angiotonin receptor blocker; CCB, calcium channel blockers; WBC, white blood cell; NEU, neutrophils; LYM, lymphocyte; RBC, red blood cell; FMR, functional mitral regurgitation.

**Table A4:** Medication Use and Laboratory Parameters of non-AFMR, mild AFMR and moderate-to-severe AFMR patients in the cohort.

	Total (*n* = 576)	Non-AFMR (*n* = 170)	Mild AFMR (*n* = 363)	Moderate-to-Severe AFMR (*n* = 43)
Medications				
ARNI, *n* (%)	177 (30.73%)	42 (24.71%)	117 (32.23%)	18 (41.86%)
ACEI/ARB, *n* (%)	88 (15.28%)	19 (11.18%)	59 (16.25%)	10 (23.26%)
β-blocker, *n* (%)	141 (24.48%)	29 (17.06%)	95 (26.17%) *	17 (39.53%) #
CCB, *n* (%)	248 (43.06%)	65 (38.24%)	160 (44.08%)	23 (53.49%)
Antidiabetic drug	199 (35.79%)	44 (26.67%)	134 (38.4%) *	21 (50.00%) #
Lipid-lowering drug	28 (4.86%)	5 (2.94%)	20 (5.51%)	3 (6.98%)
Laboratory examination				
WBC (×10^9^/L)	7.26 (3.59)	7.5 (4.13)	7.13 (3.17)	7.46 (4.57)
NEU (×10^9^/L)	5.4 (3.42)	5.6 (3.95)	5.28 (3.03)	5.56 (4.28)
LYM (×10^9^/L)	1.16 (0.68)	1.18 (0.63)	1.15 (0.72)	1.18 (0.55)
RBC (×10^12^/L)	3.03 (0.73)	3.09 (0.77)	2.99 (0.71)	3.13 (0.70)
Hemoglobin (g/L)	88.44 (21.82)	90.48 (24.95)	87.22 (20.27)	90.67 (20.92)
Platelet (×10^9^/L)	198.91 (85.21)	204.49 (96.65)	197.52 (80.04)	188.63 (79.67)
Calcium (mmol/L)	2.06 (0.35)	2.07 (0.35)	2.05 (0.33)	2.14 (0.45)
Phosphorus (mmol/L)	2.11 (0.75)	2.19 (0.77)	2.08 (0.74)	2.06 (0.73)

Continuous variables are presented as means (SD), and categorical variables are presented as counts (%). Chi-square test and one-way ANOVA test are used to compare the difference among non-AFMR, mild AFMR and moderate-to-severe AFMR patients. non-AFMR versus mild AFMR, * *p* < 0.05; non-AFMR versus moderate-to-severe AFMR, # *p* < 0.05; mild AFMR versus moderate-to-severe AFMR, Abbreviations: AF, atrial fibrillation; ACEI/ARB, angiotensin converting enzyme inhibitors/angiotonin receptor blocker; CCB, calcium channel blockers; WBC, white blood cell; NEU, neutrophils; LYM, lymphocyte; RBC, red blood cell; FMR, functional mitral regurgitation.
